# Growth–defence trade-off in rice: fast-growing and acquisitive genotypes have lower expression of genes involved in immunity

**DOI:** 10.1093/jxb/erad071

**Published:** 2023-02-25

**Authors:** Felix de Tombeur, Rémi Pélissier, Ammar Shihan, Koloina Rahajaharilaza, Florian Fort, Lucie Mahaut, Taïna Lemoine, Sarah J Thorne, Sue E Hartley, Delphine Luquet, Denis Fabre, Hans Lambers, Jean-Benoît Morel, Elsa Ballini, Cyrille Violle

**Affiliations:** CEFE, Univ Montpellier, CNRS, EPHE, IRD, Montpellier, France; School of Biological Sciences and Institute of Agriculture, The University of Western Australia, Perth, Australia; PHIM Plant Health Institute, Univ Montpellier, Institut Agro, INRAE, CIRAD, Montpellier, France; CEFE, Univ Montpellier, CNRS, EPHE, IRD, Montpellier, France; Faculty of Sciences, DS Life and Environmental Sciences, University of Antananarivo 101, Antananarivo, Madagascar; CIRAD, UMR AGAP Institut, F-34398 Montpellier, France; CEFE, Univ Montpellier, Institut Agro, CNRS, EPHE, IRD, Univ Valéry, Montpellier, France; CEFE, Univ Montpellier, CNRS, EPHE, IRD, Montpellier, France; CEFE, Univ Montpellier, CNRS, EPHE, IRD, Montpellier, France; School of Biosciences, University of Sheffield, Sheffield, UK; School of Biosciences, University of Sheffield, Sheffield, UK; CIRAD, UMR AGAP Institut, F-34398 Montpellier, France; UMR AGAP Institut, Univ Montpellier, CIRAD, INRAE, Institut Agro, Montpellier, France; CIRAD, UMR AGAP Institut, F-34398 Montpellier, France; UMR AGAP Institut, Univ Montpellier, CIRAD, INRAE, Institut Agro, Montpellier, France; School of Biological Sciences and Institute of Agriculture, The University of Western Australia, Perth, Australia; PHIM Plant Health Institute, Univ Montpellier, Institut Agro, INRAE, CIRAD, Montpellier, France; PHIM Plant Health Institute, Univ Montpellier, Institut Agro, INRAE, CIRAD, Montpellier, France; CEFE, Univ Montpellier, CNRS, EPHE, IRD, Montpellier, France; Monash University, Australia

**Keywords:** Defence gene, growth–defence trade-off, intraspecific variation, plant defence, plant economics spectrum, plant functional trait, plant immunity, rice (*Oryza sativa*), silica, silicon

## Abstract

Plant ecologists and molecular biologists have long considered the hypothesis of a trade-off between plant growth and defence separately. In particular, how genes thought to control the growth–defence trade-off at the molecular level relate to trait-based frameworks in functional ecology, such as the slow–fast plant economics spectrum, is unknown. We grew 49 phenotypically diverse rice genotypes in pots under optimal conditions and measured growth-related functional traits and the constitutive expression of 11 genes involved in plant defence. We also quantified the concentration of silicon (Si) in leaves to estimate silica-based defences. Rice genotypes were aligned along a slow–fast continuum, with slow-growing, late-flowering genotypes versus fast-growing, early-flowering genotypes. Leaf dry matter content and leaf Si concentrations were not aligned with this axis and negatively correlated with each other. Live-fast genotypes exhibited greater expression of *OsNPR1*, a regulator of the salicylic acid pathway that promotes plant defence while suppressing plant growth. These genotypes also exhibited greater expression of *SPL7* and *GH3.2*, which are also involved in both stress resistance and growth. Our results do not support the hypothesis of a growth–defence trade-off when leaf Si and leaf dry matter content are considered, but they do when hormonal pathway genes are considered. We demonstrate the benefits of combining ecological and molecular approaches to elucidate the growth–defence trade-off, opening new avenues for plant breeding and crop science.

## Teaser

Slow-growing, late-flowering (live slow–die old) rice genotypes exhibit greater expression of defence genes than fast-growing, early-flowering genotypes, demonstrating a growth–defence trade-off.

## Introduction

One of the most influential theories in plant biology is that defences against pathogens and herbivores are costly, which results in trade-offs between growth and defence (Simms and Rausher, 1987; Fagerström, 1989; Herms and Mattson, 1992). Plants have indeed developed a wide range of constitutive and induced defence mechanisms to protect themselves against pathogens and herbivores, and this comes with a cost (Agrawal, 2007; Savatin *et al.*, 2014). Plant ecologists have long considered the costs of resistance to pathogens or herbivores by testing a decrease in plant growth and/or reproduction associated with increased resistance (Strauss *et al.*, 2002; Moore *et al.*, 2003; Paul-Victor *et al.*, 2010; Garcia *et al.*, 2021). Such an approach showed some success in identifying trade-offs between growth and defence, whether chemical or mechanical (Endara and Coley, 2011; Garcia *et al.*, 2021). However, whether these trade-offs result from genetic variation or phenotypic plasticity remains unclear (Hahn and Maron, 2016; Züst and Agrawal, 2017; but see Vázquez-González *et al.*, 2020; Cope *et al.*, 2021). In addition, the emergence of trait-based ecology enabled the exploration of major spectrum shaping plant strategies, such as the *leaf economics spectrum* (LES) (Wright *et al.*, 2004), but plant mechanical or chemical defences against pathogens and herbivores are still poorly captured in this spectrum.

The LES describes a major axis of cross-species leaf ecophysiology comprising key traits such as leaf lifespan, nitrogen (N), and phosphorus (P) concentrations, and photosynthetic rate (Wright *et al.*, 2004). The spectrum runs from fast-growing species having traits associated with rapid resource acquisition and return on resource investment (acquisitive species), to slow-growing species having traits involved in resource conservation (Wright *et al.*, 2004; Reich, 2014). An implicit assumption underpinning the LES is that slow-growing species (conservative strategies) are better defended against pathogens and herbivores, to avoid the loss of scarce nutrients and hard-to-get carbon (Coley *et al.*, 1985; Endara and Coley, 2011). The implementation of the LES to study trade-offs between growth and defence or survival across different species has shown some success (Hallik *et al.*, 2009; Züst and Agrawal, 2017). For instance, conservative *Helianthus* spp. have tougher, more succulent leaves with higher tannin activity compared to more resource-acquisitive species (Mason *et al.*, 2016). However, these studies most of the time ignore trait variation within species and do not control for the role of phylogeny in shaping these patterns (Albert *et al.*, 2011). Considering the intraspecific level is therefore often more appropriate to understand mechanisms underlying the co-variations and trade-offs among traits (Vasseur *et al.*, 2012; Sartori *et al.*, 2019). In addition, although some traits thought to be involved in stress tolerance and resource conservation are often considered by trait-based ecology [e.g. leaf dry matter content (LDMC); Wright and Cannon, 2001; Mason and Donovan, 2015; Blumenthal *et al.*, 2020], others are generally ignored. For instance, mineral deposits made of silica in plant tissues can increase the resistance to herbivores (Massey and Hartley, 2009) and pathogens (Ahammed and Yang, 2021), especially in grasses including rice, but leaf silicon (Si) concentration is rarely considered in trait-based ecology (de Tombeur *et al.*, 2023).

In parallel with ecological studies, molecular biologists have long studied the plant immune system to understand how plants recognize and respond to pathogens (Ferreira *et al.*, 2006; Jones and Dangl, 2006; Karasov *et al.*, 2017). These studies allowed a thorough mechanistic understanding of the growth–defence trade-off. In particular, some genes, proteins, and hormonal pathways have been identified as important in the plant immune system and plant defence but also to mediate plant growth parameters (Ning *et al.*, 2017; van Butselaar and Van Den Ackerveken, 2020). For instance, in rice (*Oryza sativa*), the *OsNPR1* gene, originally identified as a key regulator of the salicylic acid pathway and defence response (Sugano *et al.*, 2010), also suppresses growth by interfering with the auxin pathway (Li *et al.*, 2016). Similarly, the rice protein IPA1, depending on its phosphorylation status, was shown to either activate the transcription of yield genes or to activate the defence gene *WRKY45*, leading to enhanced disease resistance (Jiao *et al.*, 2010; Wang *et al.*, 2018). These studies have been pivotal in improving our mechanistic understanding of the growth–defence trade-off (He *et al.*, 2022), but they are mostly based on one single genotype and the use of mutants, and multi-genotypic comparative studies are scarce. More generally, these studies are at the molecular level and we still ignore whether they are reflected in plant ecological strategies and associated with the LES framework, and more generally with trait-based ecology.

Here, we combine ecological and molecular approaches to study the trade-off between growth and defence/immunity at the intraspecific level. To do so, we grew 49 genotypes of temperate rice (*Oryza sativa* subsp. *japonica.*) in similar conditions and under optimal growing conditions, and we measured growth- and defence-related functional traits together with defence-related gene expression. We first built a phenotypic space based on several key traits directly involved in plant metabolism and/or the LES framework (leaf N, P, and S concentrations, chlorophyll fluorescence as a proxy for photosynthetic efficiency). Concentrations of Si—a proxy for silica-based defences—and LDMC were considered as mechanical defence/stress-resistance traits, and the leaf carbon (C):N ratio was used as a proxy for leaf nutritional quality (Agrawal and Fishbein, 2006). These traits were added to the phenotypic space to analyse the relationships between mechanical defence and LES strategies. The main axes of trait variation—and especially the one contrasting fast-growing versus slow-growing genotypes—were then compared with the expression of genes involved in basal immunity to further test the hypothesis of a growth–defence trade-off. We hypothesized that slow-growing genotypes will have higher levels of leaf mechanical defensive traits (LDMC and leaf [Si]), and will express genes involved in plant defence more strongly.

## Materials and methods

### Plant material

We selected temperate rice genotypes (*Oryza sativa* subsp. *japonica*) from a set obtained from the European Rice Germplasm Collection (Courtois *et al.*, 2012). This population was characterized for genome-wide analysis and phenotypic data were published elsewhere (Biscarini *et al.*, 2016; Volante *et al.*, 2017; Frontini *et al.*, 2021). These phenotypic data were used in a clustering analysis, and 49 lines were chosen in order to maximize phenotypic and genotypic diversity. Traits used in this analysis were root biomass, days to flowering, leaf area, panicle and node height, leaf chlorophyll concentration, flavonoid concentration, yield, hundred grain weight, and number of tillers per metre. The list of the 49 rice genotypes can be found in Supplementary Table S1.

### Growth conditions

The experiment was conducted in an experimental field of the CEFE (Montpellier, France) from June to September 2021, in outdoor conditions (mean daily temperature from 18.7 °C to 28.1 °C). We used a randomized complete block design using four blocks, with each genotype replicated once in each block (i.e. four replicates per genotype, a total of 196 pots). Plants were grown in 8.8-litre plastic pots (15 cm diameter; 50 cm depth) filled with a mixture of 50% (volume based) quartz sand and 50% soil (62% sand, 27% silt, and 11% clay) and amended with 3.5 g l^−1^ of NPK fertilizer (Basacote High K 6M NPK 13-5-18; Compo Expert) and 5.9 mg l^−1^ of Fe fertilizer (Ferveg 6; 6% Fe EDDHA). Plants were watered every day with a drip irrigation system with about 150 ml of tap water to avoid water stress.

### Plant trait measurements

Traits were measured at the beginning of the flowering stage to standardize measurements among individuals. Daily maximum and minimum temperatures were used to calculate the age at flowering in growing degree-days (GDD), as the sum of GDD from germination to the appearance of the first panicle and considering a base temperature of 10 °C (Lee *et al.*, 2015). Meteorological data were recorded using a Davis Vantage Pro2 weather station installed in the experimental field at CEFE.

The chlorophyll fluorescence, hereafter *Y*(II), was measured with a MINIPAM II (pulse amplitude modulation) fluorometer (Walz, Effeltrich, Germany). A mature N-1 leaf (below the flag leaf) was light-adjusted for approximately 5 min to reach a steady state photosynthesis level, prior to measurements of the effective quantum yield of photochemical energy conversion (yield). The relative effective quantum yield of photochemical energy conversion at steady-state photosynthesis was calculated as: yield=(*F*_m_*ʹ*−*F*_s_)/*F*_m_*ʹ* (Genty *et al.*, 1989), where *F*_s_ and *F*_m_ʹ are the fluorescence at steady-state photosynthesis and maximum fluorescence in the light, respectively. The chlorophyll fluorescence gives an estimate of the efficiency of photosystem II involved in photosynthesis. The same leaf was then collected, rehydrated overnight, weighed, and dried at 60 °C for 72 h to calculate the LDMC as the ratio between leaf dry weight and leaf fresh weight (% DW). Leaf N and C concentrations were determined on the same leaf after grinding, using a CN elemental analyser (Fisons Instruments—CHN model EA 1108).

Three additional N-1 adult leaves were then sampled on other tillers that had developed their flag leaves and begun to flower. Leaves were then dried at 60 °C for 72 h, and ground to quantify concentrations of P, Si, and S with a portable X-ray fluorescence spectrometer (Reidinger *et al.*, 2012). Dried leaf material was ball-milled (Retsch MM400 Mixer mill, Germany) and ground material was pressed at 10 tons into pellets using a manual hydraulic press with a 13 mm die (Specac, UK). Elemental analysis was performed using a P-XRF instrument (Nitron XL3t900 GOLDD analyser: Thermo Scientific, UK) held in a test stand (SmartStand, Thermo Scientific). The P-XRF instrument was calibrated using Si-spiked synthetic methyl cellulose (Sigma-Aldrich, product no. 274429) and Certified Reference Materials of NCS DC73349 ‘Bush branches and leaves’ obtained from China National Analysis Center for Iron and Steel. To avoid signal loss by air absorption, the analyses were performed under a helium atmosphere (Reidinger *et al.*, 2012). A reading of each side of the pellet was made, approximately 1 h apart, to account for u-drift in the instrument (i.e. variation in readings between consecutive runs using identical parameters; Johnson, 2014). The two readings were averaged to obtain P, Si, and S concentrations.

At harvest, aboveground biomass was dried at 60 °C for 72 h. The relative growth rate (RGR, in g g^−1^ GDD^−1^) was calculated as follows:


$$\begin{array}{l} RGR=\;\frac{ln\;W_{2}-\;ln\;W_{1}\;}{t_{2}-t_{1}} \end{array}$$

where *W*_2_ is the aboveground plant biomass at harvest, and *t*_2_–*t*_1_ is the number of GDD from germination to harvest. We used the mean weight of all the plants pulled out after germination for *W*_1_ (0.05 g).

### Gene expression

At the beginning of the flowering stage, the last fully developed leaf (flag leaf) of the tallest tiller was sampled for RNA extraction on each plant. Only the middle part of the leaves was taken. Once collected, the leaf was transferred to a tube and placed immediately in liquid nitrogen.

For RNA extraction, we used protocols described in Delteil *et al.* (2012). Briefly, frozen leaf material was ground in liquid nitrogen. Approximately 500 mg of powder was treated with 1 ml of TRIzol (Thermo Fisher Scientific, Waltham, MA, USA). RNA samples (5 µg) were denatured for 5 min at 65 °C with oligo(dT) 18 (3.5 mM) and deoxynucleoside triphosphate (dNTP) (1.5 mM). They were later subjected to reverse transcription for 45 min at 37 °C with 200 U of reverse transcriptase M-MLV (Promega, Madison, WI, USA) in the appropriate buffer.

Gene expression analysis was performed using the Fluidigm Biomark analysis protocol. Briefly, 2 µl of cDNA samples were diluted 10 times (samples around 3 µM) and pre-amplified following the ‘Pre-amplification of cDNA for Gene Expression with delta Gene Assays’ protocol provided by the manufacturer (Fluidigm). The reactions were cleaned up using Exonuclease I (Exo I, 4U μl^−1^). Gene expression analysis was performed with the 96.96 IFC Machine using the Delta Gene Assays and the protocol provided by Fluidigm on 1/10 diluted pre-amplified samples (5 µl gene assay mix and sample assays used for running the plate). Amplification was performed as follows: 95 °C for 1 min; 30 cycles of 96 °C for 5 s and 60 °C for 1 min; finally, 95 °C for 1 min. Gene expression was measured for 10 genes that were transcriptionally regulated in the leaf defence process in rice: *OsMT2*, *NPR1*, *SPL7*, *POX223*, *PBZ1*, *RBBI2*, *PR1B*, *WRKY45*, *OsCHI*, and *PR5* (Supplementary Table S2; Delteil *et al.*, 2012). We also included a gene of the *GH3* family, *GH3.2*, because of its involvement in biotic and abiotic stresses (Fu *et al.*, 2011; Du *et al.*, 2012; Kong *et al.*, 2019). Gene expression was calculated by the 2^DDCt^ method (Livak and Schmittgen, 2001) using three references genes, *EF1a*, *EF4a*, and *UBQ5*, with the R package ‘fluidigr’.

### Statistical analyses

First, we characterized the phenotypic space of the 49 genotypes using a principal component analysis (PCA) and considering three traits underlying the LES (leaf [N], leaf [P], *Y*(II)), to which we added traits related to leaf defence/stress resistance (LDMC, leaf [Si], leaf C:N ratio). Leaf [S] was added to the PCA because of its alignment with the LES framework and direct involvement in photosynthesis and growth (component of proteins) (Fratte *et al.*, 2021). RGR and life history (age at flowering) were also added to the PCA, as quantitative supplementary variables (no influence on PCA results) to consider only leaf traits in the analysis. We then ran a second PCA with gene expression, considering the genes mentioned above. PCAs were run with the package FactoMineR on genotype-mean values and log-transformed data (Lê *et al.*, 2008). Beyond PCA analyses, bivariate relations within traits and within genes were tested through standardized major axis (SMA) regressions (Warton *et al.*, 2006), using the package SMATR (Warton *et al.*, 2012).

To test potential relationships between the phenotypic space (first PCA) and the gene expression space (second PCA), genotype scores on the first two dimensions of both PCAs were extracted and potential co-variation between genotype scores on the four axes (two axes by PCAs) were tested through SMA regressions, as indicated above. We also tested potential relationships between axes of the trait PCAs and the expression of genes taken individually. All analyses were conducted in R (R Core Team, 2021).

## Results

**Fig. 1.**
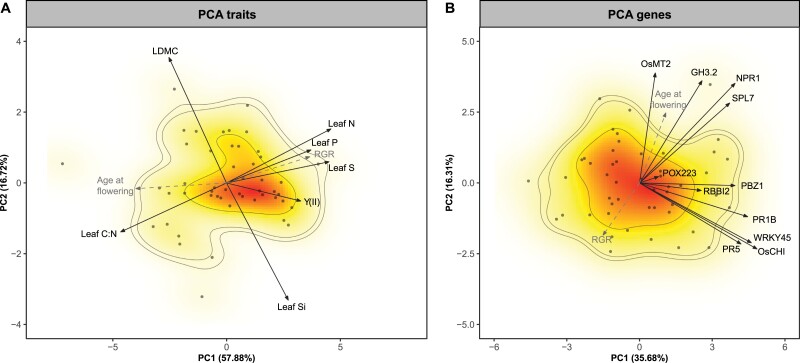
Principal component analyses (PCAs) based on correlation matrices of the main functional traits (A) and gene expression (B) for 49 rice genotypes. The relative growth rate (RGR) and age at flowering are supplementary quantitative variables for visualization (grey, dashed lines), and have no influence on the PCA results. The colour gradient indicates regions of highest (red) to lowest (white) occurrence of probability of genotypes in the traits/genes space, and contour lines show 0.50, 0.90, and 0.95 quantiles (see Díaz *et al.*, 2016 for the method). The results of the PCAs are presented in Supplementary Tables S3, S6. Both PCAs were run on log-transformed data. LDMC, leaf dry matter content; *Y*(II), chlorophyll fluorescence.

**Fig. 2.**
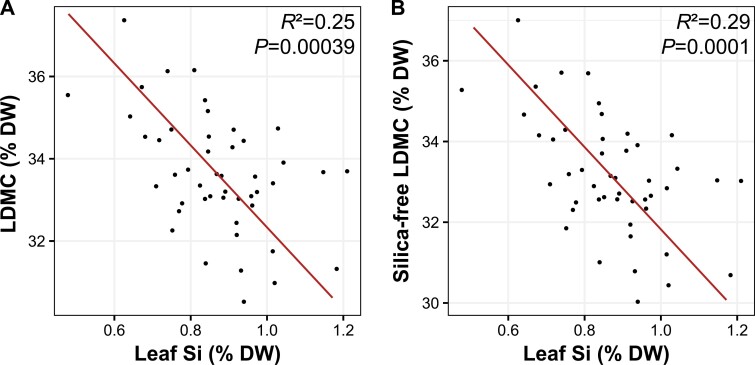
Relationship between leaf silicon concentrations and leaf dry matter content. (A) Standardized major axis regression line and statistics of the bivariate relationship between leaf silicon (Si) concentrations and leaf dry matter content (LMDC) among 49 rice genotypes. (B) To avoid spurious correlations, we also examined the relationship between leaf [Si] and silica-free LDMC. Silica-free LDMC was calculated as follows: (leaf dry weight−silica weight)/(leaf fresh weight−silica weight). The weight of silica in the leaves used for LDMC was calculated using leaf [Si]. Si was converted into silica by multiplying by 2.14 and assuming a 10% mean water content and a 5% content of other elements (Blecker *et al.*, 2006). Both relationships were very similar, with a slightly greater explanatory power for the relationship between leaf [Si] and corrected LDMC.

The first two principal components (PCs) describing the phenotypic space of rice functional traits explained 75% of the total variance (Fig. 1A; Supplementary Table S3). PC1 opposed genotypes with high foliar nutrient concentrations (leaf [N], [P], and [S]) and chlorophyll fluorescence to genotypes with higher leaf C:N ratio. The RGR and age at flowering were both well represented (Fig. 1A) and correlated (Supplementary Fig. S1) with this first axis, which overall describes a slow–fast continuum, with fast-growing, early-flowering genotypes with high nutrient concentrations contrasting with late-flowering genotypes with slower growth rates. The five traits mentioned above, RGR, and age at flowering were all significantly correlated with each other (Supplementary Table S4; Supplementary Fig. S2). Fast-growing genotypes also tended to have higher leaf [Si] but lower LDMC, although these two traits were better represented by PC2 (Fig. 1A) and negatively related to each other (either LDMC was corrected or not by silica weight; Fig. 2). Nevertheless, leaf [Si] was positively related to leaf [N], leaf [S], and *Y*(II), and negatively related to age at flowering and C:N ratio (Supplementary Table S4; Supplementary Fig. S2). Genotype-mean values of traits can be found in Supplementary Table S5.

The first two PCs describing gene expression of rice genotypes explained about 52% of the total variance (Fig. 1B; Supplementary Table S6). PC1 supported almost all genes (*NPR1*, *OsCHI*, *PBZ1*, *PR5*, *SPL7*, *WRKY45*, and *PR1B*), with the exception of *OsMT2* and *GH3.2*, which were better represented on PC2; *POX223*, which was better represented on PC4; and *RBBI2*, which was better represented on PC5 (Fig. 1B; Supplementary Table S6). Genes described by PC1 tended to be significantly correlated with each other (Supplementary Table S7). However, two groups of less-related genes were identified (*SPL7*, *NPR1*, and *GH3*.2 versus *OsCHI*, *WRKY45*, *PR5*, and *PR1B*) in the PCA (Fig. 1B) and through the correlation coefficients (Supplementary Table S7). The RGR and age at flowering were both better supported by PC2 (Fig. 1B; Supplementary Table S6), with RGR associated with lower gene expression (mostly opposed to *GH3.2*, *NPR1*, and *SPL7* expression) and age at flowering with higher expression of these same genes. Genotype-mean values of gene expression can be found in Supplementary Table S8.

**Fig. 3.**
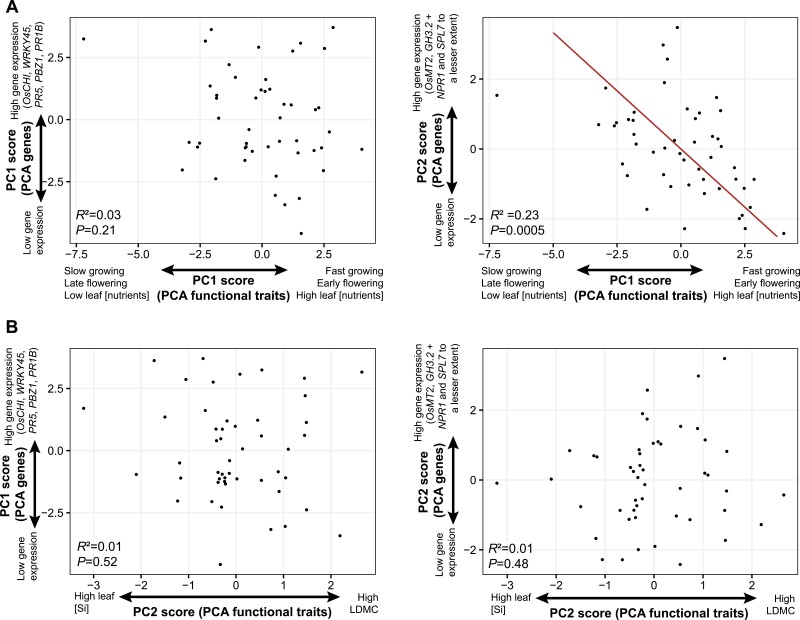
Relationships between PCA (shown in Fig. 1) axis scores. (A) Relationships between genotype scores on PC1 of the PCA considering functional traits (slow–fast continuum) and both PCs of the PCA considering gene expression. (B) Relationships between genotype scores on PC2 of the PCA considering functional traits (Si–LDMC axis) and both PCs of the PCA considering gene expression. Standardized major axis regression lines and statistics of bivariate relationships are given.

**Fig. 4.**
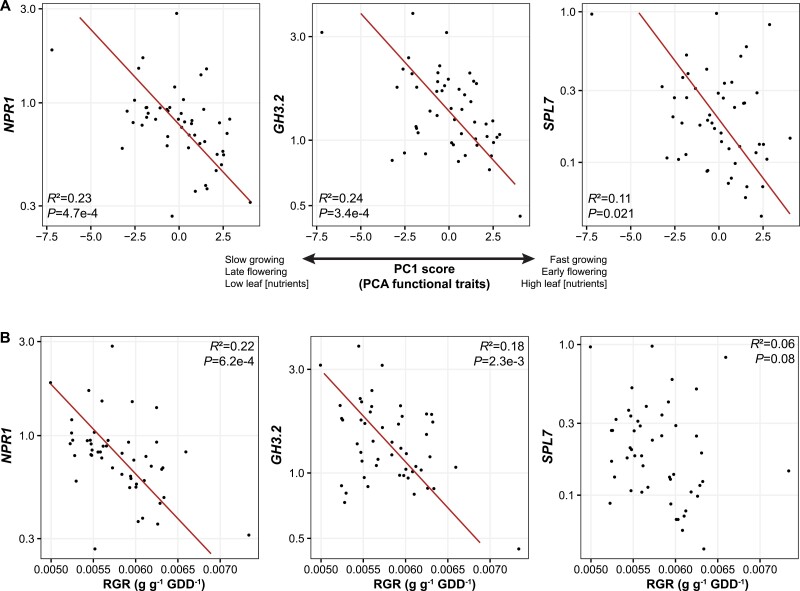
Relationships between the expression of *NPR1*, *GH3.2*, and *SPL7* and the genotype scores on PC1 of the PCA considering functional traits (slow–fast continuum) (A) and the genotypes relative growth rates (RGR) (B). Standardized major axis regression lines and statistics of bivariate relationships are given. *y*-axes are on a logarithmic scale.

Genotype scores on PC1 of the PCA considering functional traits (slow–fast continuum) were not correlated with those on PC1 of the PCA considering gene expression (Fig. 3A). However, they were negatively correlated with those on PC2 of the PCA considering gene expression (Fig. 3A), on which *OsMT2*, *GH3.2*, and, to a lesser extent, *NPR1* and *SPL7* were represented. The same observation was made when RGR was considered instead of genotype scores on PC1 of the PCA considering traits (Supplementary Fig. S3). Considering each gene individually, genotype scores on the slow–fast axis were negatively correlated with the expression of *NPR1*, *GH3.2*, and *SPL7* (Fig. 4A), but not with the expression of other genes (data not shown). The same observation was made when RGR was considered instead of PCA genotype scores (PC1), at least for *NPR1* and *GH3.2* (Fig. 4B).

Genotype scores on PC2 of the PCA considering rice functional traits (leaf [Si] versus LDMC axis) were not related to scores on PCs of the PCA genes (Fig. 3B), nor to gene expression considered individually.

## Discussion

The present study showed that ‘live slow–die old’ rice genotypes grown in optimal conditions and without biotic stress exhibited greater expression of *OsNPR1*, *OsSPL7*, and *OsGH3.2* (Figs 3, 4), which are thought to suppress growth and promote immunity in rice (Fu *et al.*, 2011; Du *et al.*, 2012; Ning *et al.*, 2017; Liu *et al.*, 2019), while fast genotypes exhibited lower expression of these genes. This result shows evidence of a growth–immunity trade-off in rice. Interestingly, this trade-off was less supported when traits considered as physical defences—leaf [Si] and leaf dry matter content (LDMC)—were considered, since these two traits were not aligned with the slow–fast continuum (Fig. 1). In fact, leaf [Si] tended to be higher among ‘live fast–die young’ genotypes. Our results also showed that, although genes involved in immunity tended to co-vary among each other, two independent molecular pathways for resistance appeared to dominate among the 49 rice genotypes (*OsNPR1* versus *OsWRKY45* (sub)pathways) (Fig. 1).

Understanding how life history strategies and the ‘slow–fast continuum’ align with key traits remains a key challenge in trait-based ecology. Here, we found strong evidence for this in a crop species, with fast-growing acquisitive and early-flowering genotypes contrasting with late-flowering genotypes with slower growth rates (Fig. 1), in accordance with other studies using non-crop species (e.g. Vasseur *et al.*, 2012; Sartori *et al.*, 2019). Interestingly, we found that leaf [S] was well-aligned with the main axis of trait variation and correlated with leaf [N], and [P], in line with interspecific trends and consistent with its role in protein synthesis (Fratte *et al.*, 2021). More surprisingly, traits considered as mechanical defences or involved in stress tolerance, such as LDMC and leaf [Si], were not aligned with this axis. Species with high LDMC are generally more resistant to stresses and have slower growth rates and decomposition rates (Blumenthal *et al.*, 2020). Its non-alignment with the LES traits was therefore somewhat surprising (e.g. Wang *et al.*, 2022), and suggests that C allocation to growth is not necessarily accompanied by less investment in structural tissues in rice.

Regarding Si, associating its concentration with either plant growth or defence is challenging because both have been demonstrated and the cost of silicification remains unclear, making potential links between leaf [Si] and key traits hard to capture (Massey *et al.*, 2007; de Tombeur *et al*., 2023). That said, leaf [Si] was correlated positively with leaf [N] and chlorophyll fluorescence, confirming global interspecific trends and suggesting a potential role of Si in photosynthesis (Detmann *et al.*, 2012; de Tombeur *et al*., 2023). Finally, the results also suggest a trade-off between LDMC and leaf [Si] among rice genotypes (Fig. 2)—in contrast to global interspecific trends where both traits are positively correlated (de Tombeur *et al*., 2023)—perhaps as different solutions to minimize the impact of some biotic and abiotic stresses and/or improving leaf mechanical properties. Interestingly, a negative relationship between leaf [Si] and leaf mass per area (LMA) has already been reported in rice (de Tombeur *et al.*, 2021), in line with the present result and reinforcing the linkages and potential trade-offs between silicification and leaf morphological traits such as LDMC, LMA, and leaf thickness (de Tombeur *et al*., 2023).

Since rice is an important crop and the most advanced model species for monocotyledonous, understanding its basal immunity level is important for several purposes (Chen and Ronald, 2011). Similar to salicylic acid concentration (Silverman *et al.*, 1995), basal immunity levels can be used as a proxy for disease resistance against several pathogens in rice (Vergne *et al.*, 2010; Grand *et al.*, 2012), but this has never been investigated in such a large intraspecific range of cultivars. Unlike in Arabidopsis, in rice defence signalling after pathogen infection branches into two gene subpathways controlled by two gene regulators, OsNPR1 and OsWRKY45 (Nakayama *et al.*, 2013; Mutuku *et al.*, 2015; van Butselaar and Van Den Ackerveken, 2020). Knockdown experiments have demonstrated that both transcriptional regulators are essential but antagonistic for induced resistance in rice. For instance, some immunity-related gene activation relies on *OsWRKY45*, but is suppressed by OsNPR1 (Ning *et al.*, 2017). Our results confirmed these two independent pathways at the intraspecific level (Fig. 1), suggesting different strategies of rice to activate defence in the *japonica* subgroup. Previously differences in the expression of *OsWRKY45* between *indica* and *japonica* subgroups were reported with different impact on rice resistance to *Xanthomonas oryzae* pv. *oryzae* (Deng *et al.*, 2018), but this regulation was not described in the *japonica* subgroup. Moreover, the link between these two pathways and trade-off regulation between growth and immunity has also been reported (van Butselaar and Van Den Ackerveken, 2020). For instance, *IPA1* (the *IDEAL PLANT ARCHITECTURE 1* gene in rice) gene can regulate either growth or immunity via *OsWRKY45* (Wang *et al.*, 2018).

Although the non-alignment of LDMC and leaf [Si] with the slow–fast axis shown in the rice phenotypic space does not support the hypothesis of a growth–defence trade-off, such a hypothesis was strongly supported by genetic data (Figs 3, 4). In particular, *OsNPR1*, *SPL7*, and *GH3.2* were more constitutively expressed among live slow–die old genotypes (Fig. 4), having more ‘conservative’ strategies. First, *OsNPR1* is well-known to both inhibit growth and enhance resistance to pathogens (Sugano *et al.*, 2010; Li *et al.*, 2016; Ning *et al.*, 2017; van Butselaar and Van Den Ackerveken, 2020). Second, *SPL7* overexpression can reduce tiller numbers in rice (Dai *et al.*, 2018) and, more generally, this gene plays a critical role in plant growth and balancing reactive oxygen species during biotic and abiotic stress (Hoang *et al.*, 2019; Liu *et al.*, 2019). Finally, *GH3.2* can mediate basal resistance by suppressing indole-3-acetic acid amido synthetase (the major form of auxin in rice) accumulation (Fu *et al.*, 2011), and is involved in growth regulation and abiotic stress resistance (Du *et al.*, 2012; Kong *et al.*, 2019). Overall, this result supports the linkage between genes identified as key regulators of the growth–immunity trade-off and traits underlying the LES/slow–fast continuum framework. It is important to note, however, that plants have been grown without fungal infection or herbivores in this study. The fact that constitutive expression of immunity-related genes is a proxy of resistance against blast fungus in rice (Grand *et al.*, 2012) suggests that the live fast–die young genotypes identified here will be more susceptible to infection, but future studies should now consider the susceptibility of these same genotypes to pathogens and herbivores. In addition to this, our results raise several other avenues for future research.

First, combining ecological and molecular approaches should be made more consistently and in different environmental conditions for a better mechanistic understanding of trait co-variation and functional trait frameworks. Ultimately, specific genes could be identified as key players in the growth–immunity trade-offs at the phenotypic level (Strauss *et al.*, 2002; Endara and Coley, 2011; Garcia *et al.*, 2021), but also more generally for trait co-variations highlighted in trait-based ecology (e.g. Díaz *et al.*, 2016). Second, how environmental parameters and trade-offs in resource allocation shape species distribution and/or population genetic variation in natural ecosystems is still not well understood (but see Cope *et al.*, 2021), and could gain from studies combining molecular and trait-based approaches. Finally, one of the key challenges in crop science is to breed varieties resistant to pathogens and herbivores, while being productive and fast-growing (Karasov *et al.*, 2017; Ning *et al.*, 2017; van Butselaar and Van Den Ackerveken, 2020; He *et al.*, 2022). On the one hand, our study clearly identified a growth–immunity trade-off that could be difficult to overcome by artificial selection. On the other hand, our multidimensional approach also identified phenotypic axes of variation that are independent of this trade-off (in particular the LDMC–leaf [Si] axis) and could be considered in breeding strategies, after assessing the benefits of these mechanical defences/stress-resistance traits. This kind of phenotype-based optimization has already been suggested in the context of plant adaptation to abiotic stresses to, for instance, overcome the drought-resistance–early growth trade-off in rice (e.g. increased growth with unchanged drought resistance through larger and thicker leaves; Rebolledo *et al.*, 2013; Luquet *et al.*, 2016). Overall, functional space approaches, such as those widely used in ecology (Mouillot *et al.*, 2021), can open new avenues for crop science and plant breeding, beyond traditional trait-by-trait approaches.

## Supplementary data

The following supplementary data are available at *JXB* online.

Fig. S1. Relationships between genotype scores on both PCs of the PCA considering functional traits (Fig. 1A) and genotypes relative growth rate (RGR) and age at flowering.

Fig. S2. Bivariate relationships between traits used to build the phenotypic space (Fig. 1A).

Fig. S3. Relationships between genotype scores on both PCs of the PCA considering gene expression (Fig. 1B) and genotype relative growth rates.

Table S1. List of the 49 rice genotypes used in this study, and countries of origin.

Table S2. IDs and origins of marker genes used for expression analysis.

Table S3. Results of the principal component analysis based on a correlation matrix of the main plant traits for 49 rice genotypes, as shown in Fig. 1A (PC1 and PC2).

Table S4. Standardized major axis statistics of bivariate relationships between the main plant traits considered in this study.

Table S5. Genotype-mean values of traits used in the principal component analysis describing the phenotypic space of rice (Fig. 1A; Supplementary Table S3).

Table S6. Results of the principal component analysis based on a correlation matrix of gene expression involved in plant defence for 49 rice genotypes, as shown in Fig. 1B (PC1 and PC2).

Table S7. Standardized major axis statistics of bivariate relationships between gene expression considered in this study.

Table S8. Genotype-mean values of relative gene expression used in the PCA (Fig. 1B; Supplementary Table S6).

erad071_suppl_Supplementary_Material

## Data Availability

All data supporting the findings of this study are available within the paper and within its supplementary data published online.
