# Correction to: Growth–defence trade-off in rice: fast-growing and acquisitive genotypes have lower expression of genes involved in immunity

**DOI:** 10.1093/jxb/erae268

**Published:** 2024-06-12

This is a correction to: Felix de Tombeur, Rémi Pélissier, Ammar Shihan, Koloina Rahajaharilaza, Florian Fort, Lucie Mahaut, Taïna Lemoine, Sarah J Thorne, Sue E Hartley, Delphine Luquet, Denis Fabre, Hans Lambers, Jean-Benoît Morel, Elsa Ballini, Cyrille Violle, Growth–defence trade-off in rice: fast-growing and acquisitive genotypes have lower expression of genes involved in immunity, Journal of Experimental Botany, Volume 74, Issue 10, 19 May 2023, Pages 3094–3103, https://doi.org/10.1093/jxb/erad071

In the published version of this article, there is an error in the following sentence in the Abstract, whereby the term “live-fast” should have been given as “live-slow”:

“Live-fast genotypes exhibited greater expression of *OsNPR1*, a regulator of the salicylic acid pathway that promotes plant defence while suppressing plant growth.”

This sentence should, therefore, read:

“Live-slow genotypes exhibited greater expression of *OsNPR1*, a regulator of the salicylic acid pathway that promotes plant defence while suppressing plant growth.”

This has been corrected only in this correction notice to preserve the published version of record.

