# Supplementary material for: Growth–defence trade-off in rice: fast-growing and acquisitive genotypes have lower expression of genes involved in immunity
Source: J Exp Bot. 2023 Feb 25;74(10):3094–103. doi: 10.1093/jxb/erad071 (PMC10199124; doi:10.1093/jxb/erad071)
Supplement: erad071_suppl_Supplementary_Material [file erad071_suppl_supplementary_material.pdf]

Supplementary Material

Growth-defence trade-off in rice: fast-growing and acquisitive genotypes have lower expression of genes involved in immunity

Journal of Experimental Botany

Felix de Tombeur[1,2,*,@], Rémi Pélissier[3,@], Ammar Shihan[1], Koloina Rahajaharilaza[4,5,6], Florian Fort[7], Lucie Mahaut[1], Taïna Lemoine[1], Sarah J. Thorne[8], Sue E. Hartley[8], Delphine Luquet[5,6], Denis Fabre[5,6], Hans Lambers[2], Jean-Benoît Morel[3], Elsa Ballini[3], Cyrille Violle[1]

[1]CEFE, Univ Montpellier, CNRS, EPHE, IRD, Montpellier, France
[2]School of Biological Sciences and Institute of Agriculture, The University of Western Australia, Perth, Australia
[3]PHIM Plant Health Institute, Univ Montpellier, Institut Agro, INRAE, CIRAD, Montpellier, France
[4]University of Antananarivo, Doctoral School Life and Environmental Sciences (ED SVE)
[5]UMR AGAP Institut, Univ Montpellier, CIRAD, INRAE, Institut Agro, Montpellier, France.
[6]CIRAD, UMR AGAP Institut, F-34398 Montpellier, France.
[7]CEFE, Université Montpellier, Institut Agro, CNRS, EPHE, IRD, Université Valéry, Montpellier, France
[8]School of Biosciences, University of Sheffield, Sheffield, United Kingdom
*Corresponding author ; felix. detombeur@cefe.cnrs.fr
@equal contribution

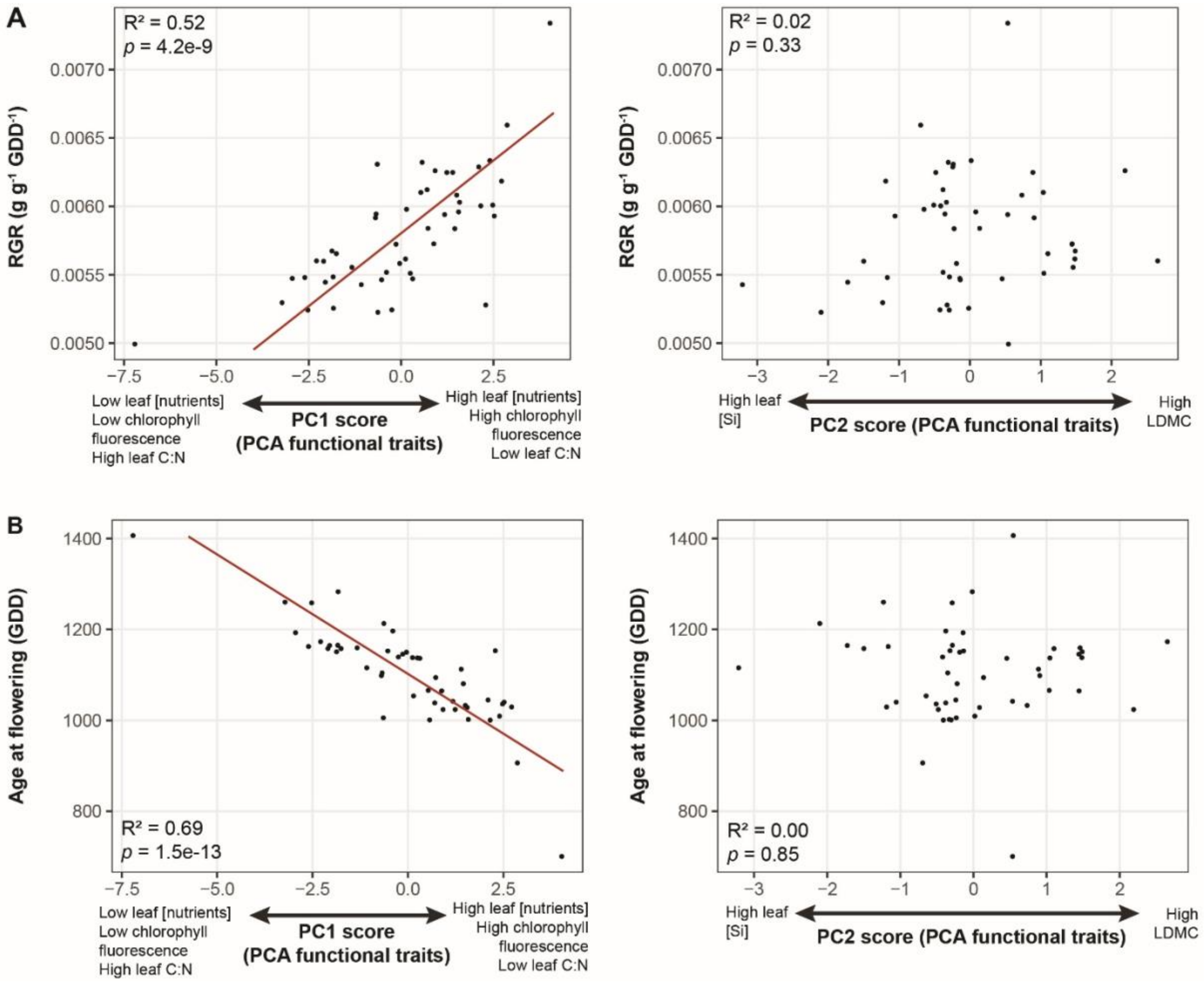

Fig. S1 – Relationships between genotype scores on both PCs of the PCA considering functional traits (Fig. 1A) and genotypes (A) relative growth rate (RGR) and (B) age at flowering. Standardised major axis (SMA) regression lines and statistics of bivariate relationships are given.

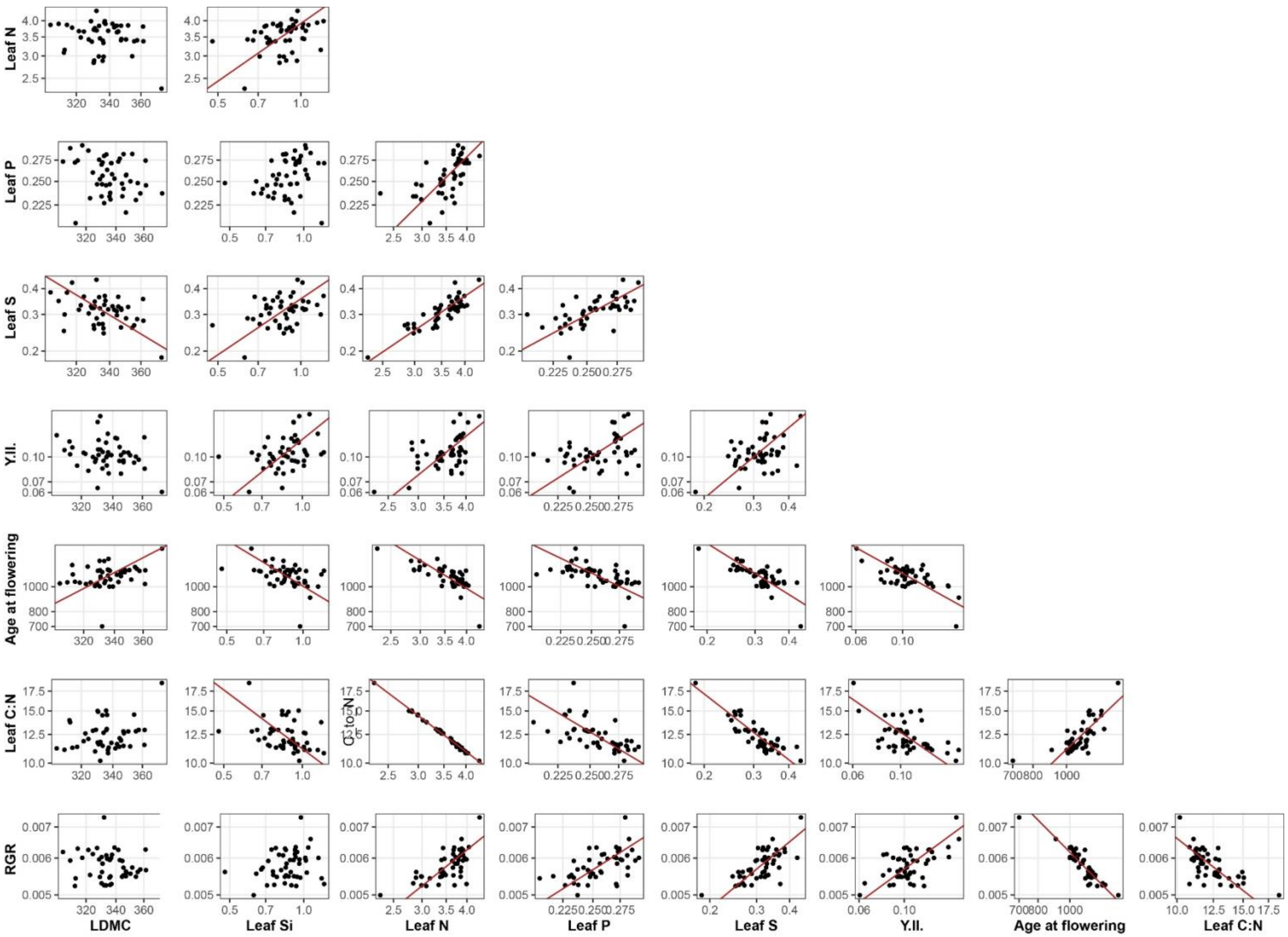

Fig. S2 – Bivariate relationships between traits used to build the phenotypic space (Fig. 1A). Standardised major axis (SMA) regression lines are shown when $p < 0.05$, and statistics of bivariate relationships are given in Table S4. Y-axes are on a logarithmic scale.

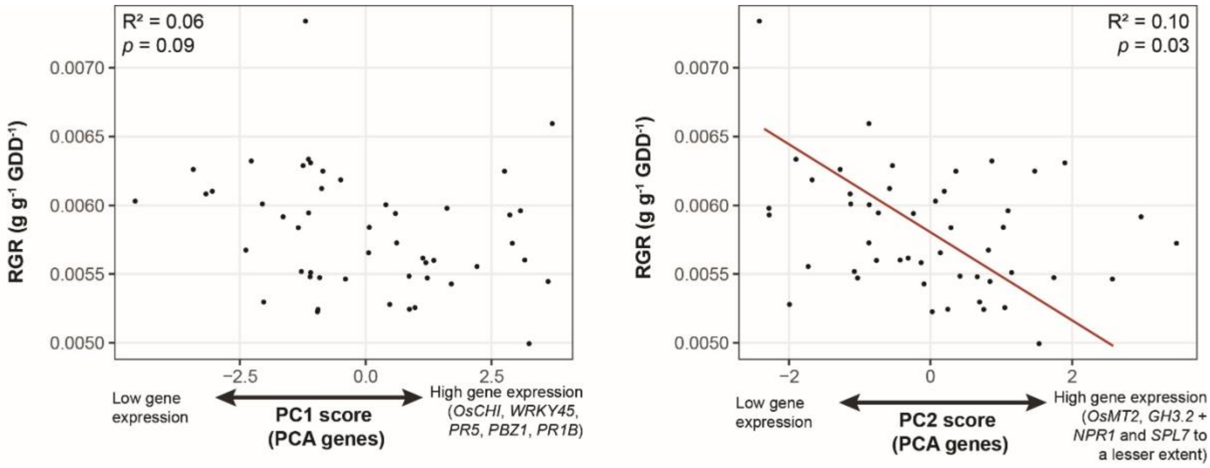

Fig. S3 – Relationships between genotype scores on both PCs of the PCA considering genes expression (Fig. 1B) and genotypes relative growth rates (RGR). Standardised major axis (SMA) regression lines and statistics of bivariate relationships are given.

Table S1 – List of the 49 rice genotypes used in this study, and countries of origin.

Genotype	Country of origin
ARELATE	France
ARIETE	Italy
AUGUSTO	Italy
BALDO 363	Italy
BARAGGIA	Italy
BRIO	Italy
CALMOCHI 101	Italy
CAPATAZ	Spain
CARINA	Bulgaria
CARNAROLI	France
CIGALON	France
CINIA 40	Chile
COLINA	Spain
ERCOLE	Italy
GAGERON	France
GINES	France
GRITNA	Italy
IBO 380/33	France
JEFFERSON	USA
KITAAKE	Japan
KORAL	Italy
KULON	Russia
LIDO	Italy
LOMELLINO	Italy
LUNA	USA
LUXOR	Italy
M202	France
M204	France
MARATELLI	Italy
MESTRE	Italy
NIPPONBARE	Japan
OPALE	Italy
OTA	Portugal
PIEMONTE	Italy
RIBE	France
RODINA	Bulgaria
S 101	USA
SAFARI	Portugal
SAVIO	Italy
SELENIO	Italy
SENIA	Spain
SESIA	Italy
SR 113	Spain
TOPAZIO	Italy
UPLA	France
VALTEJO	Portugal
VIALONE	Italy
VICTORIA	Argentina
VOLANO	Italy

Table S2 – IDs and origins of marker genes used for expression analysis.

Gene_LOC	Gene ID	Gene name	Process	Forward primer	Reverse primer	Reference
LOC_Os05g25770	Os05g0322900	*OsWRKY45*	Defense	ACGACGAGGTTGTCTTCGATCTG	GCCCGTGTCCATCCATGATTCTTC	Shimono *et al.*, 2007
LOC_Os01g09800	Os01g0194300	*OsNPR1*	Defense	CCTGATGGTTGCCTTCTGTC	ATTCAAGCACTTGTATTACACCTC	Yuan *et al.*, 2007
LOC_Os10g28080.1	Os10g0416500	*OsCHI*	Defense	TTAACGGCGCTGCTACCATT	TCCCATCCTCTTACTGCCGA	Feng *et al.*, 2011
LOC_Os01g74300	Os01g0974200	*OsMT2b*	Defense	ACCGTCGTCGTCGTTGTCAG	GCATGAGGAGATGGAGCAGGAG	Hann *et al.*, 2004
LOC_Os12g36880.1	Os12g0555500	*OsPBZ1*	Defense	CCTGCCGAATACGCCTAAGATG	AGAACACATTCAGACTTGCCTCTC	Delteil *et al.*, 2012
LOC_Os07g48020.1	Os07g48020	*OsPOX223*	Defense	CTCAGCTGCTCCAAGGTGAA	TGTGGCCCGTTTATGTCGTC	Vergne *et al.*, 2010
LOC_Os01g28450.1	Os01g28450	*OsPR1b*	Defense	CGAGAAGAGCGACTACGACTAC	GCCTCTGTCCGACGAAGTTG	Agrawal *et al.*, 2001
LOC_Os12g43430.1	Os12g0628600	*OsPR5*	Defense	AGCCAGGACTTCTACGACCT	GCGTGTGTCTTGGTGTTGTC	Delteil *et al.*, 2012
LOC_Os01g03390.1	Os01g0124650	*OsRBBI2*	Defense	GTGTGTTGTCTCGTGTGAACG	TACGATGACAGCGCAACATG	Delteil *et al.*, 2012
LOC_Os05g45410.1	Os05g0530400	*OsSPL7*	Defense	CGGATTAGAGGCTTGCGTGTTAC	GCACAGTAGTCAGCGGATAGAAC	Delteil *et al.*, 2012
LOC_Os01g55940.1	Os01g0764800	*OsGH3.2*	Defense, Auxin	AGCCTTCTACTACAACTACTACT	TGACACTGACACCGACTG	Du *et al.*, 2012

Table S3 – Results of the principal component analysis (PCA) based on a correlation matrix of the main plant traits for 49 rice genotypes, as shown in Fig. 1A (PC1 and PC2). Relative growth rate (RGR) and age at flowering are supplementary quantitative variables, and have no influence on the PCA results. Data shown are the eigenvalue, the proportion and cumulative proportion of variance explained by each principal component (PC), and the loadings of each trait. The highest loading(s) for each trait was bolded for visualisation. LDMC is leaf dry matter content and Y(II) is chlorophyll fluorescence.

	**PC1**	**PC2**	**PC3**	**PC4**
**Eigenvalue**	4.1	1.2	0.7	0.5
**Variance (%)**	57.9	16.7	9.4	7.1
**Cumulative (%)**	57.9	74.6	84.0	91.1
LDMC	-0.51	**0.71**	0.22	-0.23
Leaf Si	0.54	**-0.66**	0.02	-0.31
Leaf N	**0.92**	0.31	-0.02	-0.19
Leaf P	**0.74**	0.19	-0.17	0.50
Leaf S	**0.90**	0.12	-0.23	-0.09
Y(II)	**0.65**	-0.10	0.73	0.15
Leaf C:N	**-0.93**	-0.28	0.03	0.18
RGR	***0.73***	*0.15*	*0.14*	*0.11*
Age at flowering	***-0.80***	*-0.03*	*-0.19*	*0.01*

Table S4 – Standardised major axis (SMA) statistics of bivariate relationships between the main plant traits considered in this study. Coefficients of determination (r^2) are given in the upper right section of the matrix, and p-values in the lower left section. Significant relationships (p-values<0.05) are bolded. SMA models were run on log-transformed data. LDMC is leaf dry matter content, Y(II) is chlorophyll fluorescence and RGR is relative growth rate.

	LDMC	Leaf Si	Leaf N	Leaf P	Leaf S	Y(II)	Age at flowering	Leaf C:N	RGR
LDMC		**0.26**	0.05	0.06	**0.17**	0.08	**0.09**	0.07	0.06
Leaf Si	**2.6e-4**		**0.10**	0.08	**0.14**	**0.12**	**0.17**	**0.12**	0.06
Leaf N	0.11	**0.03**		**0.38**	**0.73**	**0.27**	**0.53**	**0.99**	**0.46**
Leaf P	0.10	0.05	**2.1e-6**		**0.39**	**0.15**	**0.31**	**0.41**	**0.33**
Leaf S	**4e-3**	**7.2e-3**	**4.7e-15**	**1.4e-6**		**0.18**	**0.54**	**0.74**	**0.49**
Y(II)	0.06	**0.01**	**1.1e-4**	**5.9e-3**	**2.2e-3**		**0.44**	**0.28**	**0.35**
Age at flowering	**0.04**	**2.8e-3**	**6.3e-9**	**3.1e-5**	**2.0e-9**	**1.7e-7**		**0.51**	**0.83**
Leaf C:N	0.08	**0.01**	**<2.2e-16**	**7.7e-7**	**2.9e-15**	**8.8e-5**	**7.1e-9**		**0.45**
RGR	0.10	0.09	**8.4e-8**	**1.5e-5**	**1.7e-8**	**6.9e-6**	**<2.2e-16**	**1.3e-7**	

Table S5 – Genotype-mean values of traits used in the PCA describing the phenotypic space of rice (Fig. 1A & Table S3). Relationships between these traits are presented in Table S4. LDMC is leaf dry matter content, Y(II) is chlorophyll fluorescence and RGR is relative growth rate.

**Genotype**	**LDMC** (mg g^{-1})	**Leaf Si** (% DW)	**Leaf N** (% DW)	**Leaf P** (% DW)	**Leaf S** (% DW)	**Y(II)**	**Age at flowering** (GDD)	**Leaf C:N**	**RGR** (g g^{-1} GDD^{-1})
ARELATE	334.0	1.02	3.46	0.26	0.32	0.09	1054	12.66	0.0060
ARIETE	330.9	0.85	3.71	0.26	0.35	0.10	1094	11.73	0.0058
AUGUSTO	337.3	0.79	3.90	0.26	0.35	0.08	1066	11.28	0.0061
BALDO 363	-	0.93	4.05	0.27	0.33	0.16	1009	10.80	0.0063
BARAGGIA	342.8	0.91	3.79	0.28	0.32	0.13	1042	11.60	0.0059
BRIO	321.5	0.92	3.67	0.29	0.32	0.11	1024	11.85	0.0062
CALMOCHI 101	330.9	0.96	3.76	0.27	0.33	0.13	1081	11.58	0.0058
CAPATAZ	336.1	0.76	3.34	0.24	0.26	0.09	1283	13.04	0.0053
CARINA	357.4	0.67	3.41	0.24	0.29	0.10	1151	12.95	0.0057
CARNAROLI	335.7	0.97	2.88	0.23	0.27	0.12	1165	15.05	0.0054
CIGALON	-	1.07	3.86	0.28	0.35	0.19	906	11.07	0.0066
CINIA 40	355.5	0.48	3.38	0.25	0.27	0.10	1173	12.82	0.0056
COLINA	344.4	0.94	3.84	0.25	0.31	0.09	1136	11.36	0.0055
ERCOLE	332.0	0.89	3.69	0.23	0.29	0.11	1139	11.79	0.0052
GAGERON	333.3	0.71	2.99	0.25	0.27	0.08	1258	14.51	0.0052
GINES	347.1	0.75	3.84	0.26	0.32	0.09	1138	11.43	0.0056
GRITNA	309.8	1.02	3.90	0.29	0.35	0.11	1040	11.09	0.0059
IBO 380/33	336.3	0.87	2.98	0.23	0.24	0.10	1162	14.55	0.0055
JEFFERSON	312.8	0.93	3.08	0.27	0.25	0.13	1213	13.96	0.0052
KITAAKE	331.9	0.97	4.34	0.28	0.44	0.18	701	10.18	0.0073
KORAL	333.5	0.82	3.83	0.28	0.36	0.11	1033	11.44	0.0061
KULON	317.5	1.02	3.78	0.29	0.43	0.09	1036	11.35	0.0060
LIDO	345.3	0.68	3.66	0.24	0.37	0.10	1146	11.98	0.0057
LOMELLINO	336.7	1.15	3.94	0.27	0.33	0.14	1000	11.07	0.0060
LUNA	361.5	0.81	3.38	0.25	0.28	0.08	1158	12.95	0.0057
LUXOR	335.8	0.88	3.40	0.25	0.30	0.10	1104	12.94	0.0059
M202	347.1	0.91	3.41	0.22	0.26	0.10	1165	12.81	0.0055
M204	350.3	0.64	3.43	0.25	0.29	0.11	1159	12.80	0.0056
MARATELLI	361.3	0.74	3.81	0.27	0.36	0.13	1024	11.52	0.0063
MESTRE	322.6	0.75	3.49	0.23	0.33	0.10	1197	12.60	0.0055
NIPPONBARE	373.7	0.63	2.30	0.24	0.19	0.06	1407	18.66	0.0050
OPALE	324.4	0.92	3.66	0.26	0.37	0.08	1038	12.07	0.0061
OTA	330.2	0.84	2.84	0.23	0.27	0.06	1260	15.01	0.0053
PIEMONTE	305.2	0.94	3.87	0.27	0.38	0.14	1029	11.22	0.0062
RIBE	347.4	1.03	3.45	0.26	0.32	0.11	1150	12.61	0.0056
RODINA	330.3	0.93	3.72	0.27	0.32	0.17	1002	11.74	0.0060
S 101	345.4	0.85	3.67	0.28	0.33	0.08	1137	11.91	0.0055
SAFARI	314.6	0.84	3.87	0.27	0.38	0.11	1045	11.26	0.0063
SAVIO	341.8	0.85	3.90	0.27	0.33	0.13	1113	11.21	0.0062
SELENIO	328.6	0.96	3.99	0.28	0.33	0.09	1028	10.95	0.0060
SENIA	337.0	1.21	3.99	0.27	0.37	0.11	1153	10.80	0.0053
SESIA	329.2	0.78	3.37	0.25	0.32	0.10	1153	12.96	0.0055
SR 113	313.2	1.18	3.15	0.21	0.30	0.10	1116	13.74	0.0054
TOPAZIO	327.2	0.77	3.43	0.25	0.30	0.10	1005	12.87	0.0063
UPLA	354.2	0.84	2.99	0.23	0.26	0.09	1193	14.58	0.0055
VALTEJO	344.5	0.72	3.64	0.23	0.30	0.12	1098	12.13	0.0059
VIALONE	339.1	1.04	3.68	0.25	0.32	0.10	1001	11.71	0.0063
VICTORIA	351.6	0.84	3.85	0.28	0.33	0.10	1065	11.38	0.0057
VOLANO	330.5	0.89	2.89	0.25	0.26	0.11	1158	14.91	0.0056

Table S6 – Results of the principal component analysis (PCA) based on a correlation matrix of gene expressions involved in plant defence for 49 rice genotypes, as shown in Fig. 1B (PC1 and PC2). Relative growth rate (RGR) and age at flowering are supplementary quantitative variables, and have no influence on the PCA results. Data shown are the eigenvalue, the proportion and cumulative proportion of variance explained by each principal component (PC), and the loadings of each trait. The highest loading(s) for each trait was bolded for visualisation.

	**PC1**	**PC2**	**PC3**	**PC4**	**PC5**
**Eigenvalue**	3.9	1.8	1.3	1.3	0.9
**Variance (%)**	35.7	16.3	11.5	11.5	8.2
**Cumulative (%)**	35.7	52.0	63.5	75.0	83.2
NPR1	**0.66**	0.59	0.20	-0.11	-0.09
OsCHI	**0.81**	-0.39	0.32	-0.17	0.11
OsMT2	0.11	**0.65**	0.15	0.34	0.41
PBZ1	**0.66**	-0.02	-0.66	0.06	-0.08
POX223	0.13	0.04	0.22	**0.86**	-0.24
PR5	**0.70**	-0.36	0.00	0.48	-0.11
RBBI2	0.42	-0.04	-0.29	0.11	**0.72**
SPL7	**0.62**	0.47	-0.44	-0.04	-0.17
WRKY45	**0.77**	-0.35	0.44	-0.13	0.15
GH3.2	0.43	**0.60**	0.36	-0.23	-0.13
PR1B	**0.74**	-0.20	-0.13	-0.19	-0.23
RGR	*-0.25*	***-0.31***	*-0.14*	*0.21*	*0.22*
Age at flowering	*0.18*	***0.41***	*0.14*	*-0.25*	*-0.23*

Table S7 – Standardised major axis (SMA) statistics of bivariate relationships between gene expressions considered in this study. Coefficients of determination (r^2) are given in the upper right section of the matrix, and p-values in the lower left section. Significant relationships (p-values<0.05) are bolded. SMA models were run on log-transformed data.

	NPR1	*OsCHI*	*OsMT2*	*PBZ1*	*POX223*	*PR5*	*RBBI2*	*SPL7*	*WRKY45*	*GH3.2*	*PR1B*
NPR1		**0.14**	**0.12**	**0.08**	0.01	0.03	0.02	**0.36**	**0.15**	**0.39**	0.09
OsCHI	**0.008**		0.01	**0.11**	0.00	**0.34**	0.08	0.04	**0.94**	0.04	**0.32**
OsMT2	**0.01**	0.53		0.00	0.02	0.00	0.01	0.04	0.00	0.06	0.04
PBZ1	**0.04**	**0.02**	0.92		0.00	**0.27**	**0.11**	**0.41**	0.05	0.00	**0.27**
POX223	0.51	0.92	0.37	0.85		**0.17**	0.00	0.00	0.00	0.00	0.00
PR5	0.24	**1.2e-5**	0.83	**1.5e-4**	**0.003**		0.05	0.06	**0.34**	0.00	**0.24**
RBBI2	0.36	0.05	0.50	**0.02**	0.80	0.13		0.05	0.06	0.00	0.08
SPL7	**5.6e-6**	0.15	0.19	**7.6e-7**	0.88	0.09	0.12		0.02	**0.13**	**0.09**
WRKY45	**0.006**	**<2.2e-16**	0.79	0.12	0.90	**1.0e-5**	0.08	0.28		0.05	**0.20**
GH3.2	**1.5e-06**	0.19	0.09	0.84	0.77	0.93	0.64	**0.01**	0.11		0.05
PR1B	0.05	**4.9e-05**	0.20	**2.3e-04**	0.93	**7.0e-04**	0.05	**0.04**	**2.2e-03**	0.12	

Table S8 – Genotype-mean values of relative gene expression used in the PCA (Fig. 1B & Table S6). Relationships between these traits are presented in Table S7. Gene expression was calculated by the 2−ΔΔCt method (Livak and Schmittgen, 2001) using three references genes, EF1a, EF4a and UBQ5, with the R package "*fluidigr*".

**Genotype**	*NPR1*	*OsCHI*	*OsMT2*	*PBZ1*	*POX223*	*PR5*	*RBBI2*	*SPL7*	*WRKY45*	*GH3.2*	*PR1B*
ARELATE	0.75	0.63	3.30	0.010	4.37	3.09	0.21	0.14	0.66	0.97	0.00020
ARIETE	0.81	0.14	9.77	0.021	0.42	0.15	0.51	0.34	0.14	1.39	0.00004
AUGUSTO	0.59	0.04	7.83	0.001	0.88	0.10	0.96	0.07	0.03	0.80	0.00001
BALDO 363	0.49	0.34	6.04	0.002	1.65	0.34	0.49	0.04	0.38	0.85	0.00001
BARAGGIA	0.62	0.24	6.80	0.005	3.21	0.79	0.50	0.13	0.25	2.04	0.00007
BRIO	1.39	0.49	5.93	0.041	1.44	0.58	2.00	0.51	0.50	1.50	0.00013
CALMOCHI 101	0.64	0.04	3.69	0.006	1.39	0.28	0.48	0.25	0.04	1.21	0.00002
CAPATAZ	0.95	0.19	13.37	0.011	1.93	0.75	0.26	0.27	0.20	1.75	0.00008
CARINA	0.95	0.04	5.80	0.002	0.46	0.14	0.06	0.11	0.04	1.16	0.00003
CARNAROLI	1.69	0.71	5.28	0.023	1.82	1.01	0.13	0.37	0.67	3.84	0.01575
CIGALON	0.83	0.63	4.84	0.499	0.75	0.78	1.69	0.82	0.25	1.06	0.20909
CINIA 40	1.50	0.64	4.54	0.095	0.86	0.50	0.89	0.10	0.59	2.40	0.03534
COLINA	0.80	0.25	2.29	0.024	2.02	0.91	0.66	0.34	0.27	1.24	0.00006
ERCOLE	1.20	0.47	8.01	0.000	1.13	0.28	0.40	0.17	0.49	1.78	0.00220
GAGERON	1.03	0.07	6.03	0.007	0.62	0.11	1.04	0.27	0.09	0.87	0.00001
GINES	0.88	0.18	6.38	0.007	5.87	1.78	0.98	0.16	0.16	1.40	0.00014
GRITNA	0.54	0.40	8.06	0.176	1.01	6.14	1.12	0.13	0.45	0.98	0.03876
IBO 380/33	0.79	0.07	3.03	0.008	0.77	0.22	0.08	0.20	0.07	2.13	0.00002
JEFFERSON	0.91	0.17	3.27	0.001	0.53	0.17	0.41	0.09	0.17	2.04	0.00001
KITAAKE	0.31	0.13	4.72	0.020	1.34	0.95	0.77	0.14	0.14	0.45	0.00000
KORAL	0.38	0.07	2.69	0.001	0.91	0.05	0.84	0.06	0.07	1.01	
KULON	0.57	0.06	1.98	0.000	2.23	0.15	0.26	0.07	0.07	1.23	0.00055
LIDO	2.84	0.16	12.74	0.024	1.59	0.47	1.77	0.98	0.16	3.13	0.00007
LOMELLINO	0.55	0.22	5.39	0.012	3.22	1.25	0.17	0.29	0.19	1.04	0.00006
LUNA	0.89	0.10	3.92	0.030	1.40	0.50	0.12	0.39	0.08	1.08	0.00015
LUXOR	0.61	0.16	3.97	0.001	0.81	0.15	0.67	0.09	0.15	1.30	
M202	0.84	0.04	5.40	0.024	3.88	1.79	0.67	0.52	0.05	1.13	0.00077
M204	0.82	0.45	2.13	0.437	1.11	0.61	0.75	0.31	0.10	0.87	0.08072
MARATELLI	0.36	0.04	2.21	0.001	0.91	0.18	0.13	0.10	0.04	0.85	0.00001
MESTRE	0.27	0.06	2.28	0.012	0.48	0.31	1.90	0.18	0.05	1.69	0.00005
NIPPONBARE	1.86	0.83	5.46	0.004	1.22	0.28	0.26	0.96	0.77	3.14	
OPALE	0.78	0.23	6.89	0.002	1.88	0.40	0.12	0.08	0.22	1.07	0.00001
OTA	0.59	0.04	5.39	0.031	2.23	0.08	0.17	0.32	0.03	0.81	0.00001
PIEMONTE	0.70	0.14	1.70	0.001	7.66	0.63	0.76	0.10	0.13	1.03	0.00005
RIBE	0.83	0.24	3.47	0.020	1.38	0.54	0.76	0.29	0.24	1.91	0.00008
RODINA	0.37	0.02	5.26	0.000	0.98	0.06	0.09	0.07	0.02	0.95	0.00001
S 101	1.04	0.04	7.24	0.026	1.77	0.35	0.12	0.27	0.04	0.95	0.00001
SAFARI	0.45	0.13	8.13	0.002	1.68	0.68	0.15	0.12	0.12	1.19	0.00002
SAVIO	0.93	0.04	5.10	0.015	1.62	0.27	0.29	0.25	0.04	1.87	0.00002
SELENIO	1.49	0.36	8.49	0.051	2.39	0.75	3.35	0.59	0.35	1.82	0.00016
SENIA	0.79	0.25	2.74	0.011	3.47	1.53	0.32	0.13	0.24	0.73	0.00008
SESIA	0.95	0.03	18.64	0.015	2.61	0.59	0.53	0.21	0.02	2.20	0.00006
SR 113	0.94	0.56	12.00	0.019	1.74	0.76	0.79	0.18	0.58	1.36	0.00004
TOPAZIO	0.67	0.04	14.11	0.003	5.69	0.22	0.60	0.19	0.04	1.86	
UPLA	0.91	0.04	15.68	0.001	6.36	0.31	1.05	0.11	0.05	1.75	0.00005
VALTEJO	0.93	0.01	10.33	0.029	2.25	0.11	0.32	0.42	0.02	1.68	0.00001
VIALONE	0.69	0.06	4.85	0.000	2.41	0.09	0.20	0.12	0.05	1.72	0.00001
VICTORIA	0.69	0.10	1.47	0.035	2.30	0.30	0.59	0.23	0.09	1.62	0.00729
VOLANO	0.77	0.21	3.02	0.011	2.36	1.54	0.43	0.18	0.22	2.02	0.00057

References

Agrawal GK, Rakwal R, Jwa NS. 2001. Differential induction of three pathogenesis-related genes, *PR10*, *PR1b* and *PR5* by the ethylene generator ethephon under light and dark in rice (*Oryza sativa* L.) seedlings. Journal of Plant Physiology **158**, 133–137.

Delteil A, Blein M, Faivre-Rampant O, Guellim A, Estevan J, Hirsch J, Bevitori R, Michel C, Morel JB. 2012. Building a mutant resource for the study of disease resistance in rice reveals the pivotal role of several genes involved in defence. Molecular Plant Pathology **13**, 72–82.

Du H, Wu N, Fu J, Wang S, Li X, Xiao J, Xiong L. 2012. A GH3 family member, OsGH3-2, modulates auxin and abscisic acid levels and differentially affects drought and cold tolerance in rice. Journal of Experimental Botany **63**, 6467–6480.

Feng JX, Cao L, Li J, *et al.* 2011. Involvement of OsNPR1/NH1 in rice basal resistance to blast fungus *Magnaporthe oryzae*. European Journal of Plant Pathology **131**, 221–235.

Hann LW, Sakamoto T, Kawasaki T, Umemura K, Shimamoto K. 2004. Down-regulation of metallothionein, a reactive oxygen scavenger, by the small GTPase OsRac1 in rice. Plant Physiology **135**, 1447–1456.

Livak KJ, Schmittgen TD. 2001. Analysis of relative gene expression data using real-time quantitative PCR and the 2-ΔΔCT method. Methods **25**, 402–408.

Shimono M, Sugano S, Nakayama A, Jiang CJ, Ono K, Toki S, Takatsuji H. 2007. Rice WRKY45 plays a crucial role in benzothiadiazole-inducible blast resistance. Plant Cell **19**, 2064–2076.

Vergne E, Grand X, Ballini E, Chalvon V, Saindrenan P, Tharreau D, Nottéghem JL, Morel JB. 2010. Preformed expression of defense is a hallmark of partial resistance to rice blast fungal pathogen *Magnaporthe oryzae*. BMC Plant Biology **10**.

Yuan Y, Zhong S, Li Q, *et al.* 2007. Functional analysis of rice *NPR1*-like genes reveals that *OsNPR1/NH1* is the rice orthologue conferring disease resistance with enhanced herbivore susceptibility. Plant Biotechnology Journal **5**, 313–324.
